# Modulation of Gut Microbes and Hepatic Metabolites by PCP Ameliorates NASH and Fatigue-like Performance in Mice †

**DOI:** 10.3390/nu17233797

**Published:** 2025-12-03

**Authors:** Yanyan Hong, Jianmei Yang, Yuanfei Wang, Dongliang Chen, Aiping Wu, Minhui Li, Wanyi Ou, Guiru Lin, Chenli Lin, Yinji Liang

**Affiliations:** 1 School of Nursing, Jinan University, Guangzhou 510632, China; 18659403749@163.com (Y.H.); yjm6782022@163.com (J.Y.); yuanfei_wang526@163.com (Y.W.); chendongliang@gzucm.edu.cn (D.C.); 17877723256@163.com (A.W.); 18729001131@163.com (M.L.); owy15625565622@163.com (W.O.); gtr201820@163.com (G.L.); 2 School of Medicine, Jinan University, Guangzhou 510632, China; 3 Health Science Center, Jinan University, Guangzhou 510632, China

**Keywords:** *Poria cocos polysaccharide*, non-alcoholic steatohepatitis, microbiome, metabolomics, fatigue-like performance

## Abstract

**Background/Objectives**: Non-alcoholic steatohepatitis (NASH) is a progressive liver condition closely associated with gut microbial dysbiosis and hepatic metabolic abnormalities. *Poria cocos polysaccharide* (PCP), a bioactive component derived from the medicinal fungus *Poria cocos*, possesses hepatoprotective properties, yet the therapeutic mechanisms of PCP in NASH, particularly those involving microbial and metabolic regulation, remain incompletely elucidated. This study aimed to investigate the effects of PCP on improving NASH and explore its mechanisms related to prebiotic activity. **Methods**: Mice were induced to develop NASH using a Western diet, followed by PCP intervention for 12 weeks. Hepatic function, including liver enzymes and lipids, glucose metabolism, and liver histopathological changes, was assessed. Fatigue and neurobehavioral alterations were evaluated via rotarod, open field, and tail suspension tests. Hepatic pro-inflammatory cytokines were measured using RT-qPCR. Gut microbiota were analyzed through 16S RNA gene sequencing, and metabolites of liver tissue were analyzed through untargeted metabolomics. **Results**: PCP decreased blood glucose and hepatic lipid levels in NASH mice, alleviating liver inflammation, ballooning degeneration, and fibrosis. It also improved fatigue-like performance on rotarod test and reduced the hepatic expression of IL-6, IL-1β, TNF-α, and IL-18. Microbiota analysis revealed that PCP restored gut microbial diversity, promoted the growth of beneficial taxa such as *Alistipes* and *Butyricoccaceae_UCG-009*, and inhibited harmful bacteria, including *Romboutsia ilealis*. Liver metabolomics showed that PCP normalized key metabolites like taurocholate and regulated taurine and hypotaurine metabolism, which were correlated with reduced inflammation, fatigue-like performance, and fibrosis. **Conclusions**: PCP, as a promising edible agent, alleviates hepatic damage, metabolic disorders, and fatigue-like performance on rotarod test in NASH mice, probably by reshaping gut microbiota and modulating hepatic taurine and hypotaurine metabolism.

## 1. Introduction

Non-alcoholic steatohepatitis (NASH), an advanced stage of non-alcoholic fatty liver disease, is defined by a triad of pathological features: liver fat accumulation exceeding 5% of hepatocytes, inflammatory activity, and evidence of hepatocyte damage. This condition frequently advances through stages of hepatic fibrosis and cirrhosis, and can ultimately result in hepatocellular carcinoma (HCC) [1]. Its pathogenesis involves multifactorial interactions, including insulin resistance, dysregulated lipid metabolism, oxidative stress, and gut dysbiosis [2]. NAFLD affects 25–30% of the global population, with 20–30% progressing to NASH. The growing prevalence of obesity, type 2 diabetes, and metabolic syndrome has precipitated a significant surge in NASH cases. Consequently, this condition has become one of the foremost causes of chronic liver pathology, especially within developed countries and areas undergoing rapid urbanization [3]. Projections suggest NASH-related cirrhosis will affect 105 million people by 2030, making it a key driver of rising liver transplantation demand [4]. NASH development is linked to high-fat, high-sugar diets and sedentary lifestyles, with consequent gut dysbiosis and metabolic disturbances identified as key risk factors [5].

Plant polysaccharides are polymers of >10 monosaccharides linked by glycosidic bonds. Abundant in diverse plant sources, they are recognized as important natural bioactive compounds. They exhibit linear, branched, or cyclic structures, with molecular weights ranging from several thousand to millions of Da, and their composition and molecular weight vary across species [6]. Resistant to direct gut digestion and absorption, they are often metabolized by gut microbiota as prebiotics [6,7]. Thus, the intake of polysaccharides can modulate gut microbiota and metabolic pathways. Mounting evidence supports their immunomodulatory, antioxidant, anti-inflammatory, metabolic regulatory, and gut microbiota-modulating effects. For example, Ganoderma lucidum polysaccharides and chitosans reduce hepatic lipids and inflammatory markers to alleviate hyperlipidemia in murine models [8]; Lycium barbarum polysaccharides (LBP) delay hepatic stellate cell (HSC) activation and prevent liver fibrosis by upregulating Smad7, a negative regulator of TGF-β/Smad signaling [9]. These findings highlight their substantial medicinal potential.

*Poria cocos*, a medicinal fungus in the *Polyporaceae* family, is a key component of the traditional formula Ginseng Ling Bai Zhu San, valued for regulating spleen-stomach function and hepatoprotection. First documented in Shen Nong Ben Cao Jing, its primary component, *Poria cocos polysaccharide* (PCP), has recently garnered extensive research attention. PCP is a highly branched reticular molecule with a β-(1→3)-glucan backbone and β-(1→6)-glucan side chains [10]. It includes water-soluble and alkali-soluble fractions; the water-soluble fraction primarily contains glucose, mannose, galactose, and fucose. PCP exhibits multiple bioactivities, including immunomodulation, anti-inflammatory effects, hepatoprotection, and antidepressant properties [10,11]. Animal research indicates that PCP offers multiple therapeutic benefits. In immunocompromised mice, it stimulates the growth of splenic lymphocytes and provides anti-inflammatory and antioxidant benefits by suppressing COX-2, iNOS, TNF-α, and IL-6 [12,13]. Intestinal barrier integrity is another target of PCP action, which modulates the gut microbiota by promoting beneficial genera (e.g., *Bifidobacterium* and *Lactobacillus*) and inhibiting pathogenic ones (e.g., *Escherichia coli*) [14]. PCP also addresses metabolic disorders; in obese mice, it reduces insulin resistance. This is achieved through gut microbiota-derived short-chain fatty acids (SCFAs) that activate the FGF21/PI3K/AKT signaling pathway [15]. However, prior research has focused mainly on PCP’s immunomodulatory activity and role in maintaining intestinal barrier function. Given its low toxicity and multi-target profile, PCP holds promise as a natural NASH intervention. Carbon tetrachloride (CCL_4_) has been widely used for decades to induce liver fibrosis in mice. Studies have shown that a Western diet combined with intraperitoneal injections of trace amounts of CCL_4_ can shorten the time required to establish a NASH mouse model to 12 weeks [16]. Thus, building upon our previous research, this study will establish a NASH mouse model using a Western diet combined with intraperitoneal injections of trace amounts of CCL_4_, and employ PCP intervention to explore how PCP ameliorates NASH by modulating gut microbiota and hepatic metabolites [17].

## 2. Materials and Methods

### 2.1. Extraction and Analysis of Poria cocos polysaccharides

The *Poria cocos polysaccharide* (PCP) sample, with a certified purity of ≥98%, was supplied by Shanxi Ciyuan Biotechnology Co., Ltd. (Xi’an, China; Batch No. CY190105). Monosaccharide composition of PCP was analyzed using a Thermo ICS 5000+ ion chromatography system (Thermo Fisher Scientific, Waltham, MA, USA) equipped with a Dionex™ CarboPac™ PA20 column (Thermo Fisher Scientific, Waltham, MA, USA) (150 × 3.0 mm, 10 μm), with a flow rate of 0.5 mL/min and column temperature of 30 °C. Mobile phases were water (A), 0.1 M NaOH (B), and 0.1 M NaOH/0.2 M NaAc (C). The gradient elution program was as follows: 0 min, 95:5:0 (A:B:C); 26 min, 85:5:10; 42 min, 85:5:10; 42.1 min, 60:0:40; 52 min, 60:40:0; 52.1 min, 95:5:0; equilibrated to 60 min. Solid samples were hydrolyzed with 2 M TFA at 121 °C for 2 h, then dried under nitrogen, washed, and re-dissolved in methanol. Liquid samples were concentrated and processed identically. A 10 mg/mL standard stock solution was serially diluted to prepare a mixed standard series (60, 50, or 40 μg/mL), with an injection volume of 5 μL. Analyses were performed using an electrochemical detector (Thermo Fisher Scientific, Waltham, MA, USA). Fresh samples were decolorized with ethanol, water-extracted, and the centrifugation residue was processed as per liquid samples [18]. Fourier transform infrared (FTIR) spectroscopy (Nicolet iZ-10, Thermo, Waltham, MA, USA) was used to identify PCP’s functional groups.

### 2.2. Animal Care and Experimental Design

All experimental procedures received approval from the Institutional Animal Care and Use Committee (IACUC) of Jinan University, China (No. IACUC-20220301-17). 6–8-week-old male C57BL/6J mice were obtained from Zhejiang Weitong Lihua Laboratory Animal Technology Co., Ltd. (Beijing, China). The mice were maintained in the Animal Center of Jinan University under standardized conditions, which included a temperature of 23 ± 2 °C, 55% ± 5% humidity, and a 12-h photoperiod. Food and water were provided ad libitum [19]. All experiments were conducted following guidelines approved by the Institutional Animal Care and Use Committee (IACUC) of Jinan University, China (No. IACUC-20220301-17). Mice were randomly assigned to three groups (*n* = 5 per group): ND group (normal diet, no CCl_4_ injection), WDC group (Western diet, intraperitoneal injections of trace amounts of CCL_4_), and WDC_PCP group (Western diet, intraperitoneal injections of trace amounts of CCL_4_ + PCP treatment). The ND group received a standard laboratory chow (5C02). The WDC and WDC_PCP groups were fed a Western diet (TP.120528A, Nantong Tianjian Animal Feed Hi-Tech Co., Ltd., Nantong, China) with the following composition (by weight): 17.3% protein, 48.5% carbohydrates, 1.25% cholesterol, and 21.2% fat. All groups had access to daily drinking water, with the non-control groups additionally provided a high-sugar solution (23.1 g/L d-fructose and 18.9 g/L d-glucose; F108334, G116306, Aladdin, Shanghai, China) while the control group received pure water only. To induce NASH, the WDC and WDC_PCP groups received weekly intraperitoneal injections of CCl_4_ (0.2 μL (0.32 μg)/g of body weight; Sinopharm Chemical Reagent Co., Ltd., 10006464, Shanghai, China) dissolved in corn oil (10% CCl_4_/corn oil) [16]. The ND group received weekly intraperitoneal injections of an equal volume of corn oil as a control. The WDC_PCP group was treated with PCP (236 mg/kg) via daily gavage for 12 weeks; the ND and WDC groups received an equal volume of distilled water via daily gavage. Body weight and food intake were recorded weekly [20]. At week 12, feces were collected from all mice and stored at −80 °C. Blood was then collected via orbital sinus puncture after isoflurane anesthesia, allowed to stand for 2 h, and centrifuged at 1200 rpm for 10 min at 4 °C. The serum supernatant was harvested and stored at −80 °C. Mice were humanely euthanized by cervical dislocation according to the approved animal care and use protocol, followed by collection and weighing of liver tissue. Liver samples were rinsed with saline, with portions fixed in 4% paraformaldehyde (BL539A, Biosharp, Hefei, China) and the remainder stored at −80 °C.

### 2.3. Behavioral Tests

#### 2.3.1. Open Field Test (OFT)

Mice were tested in a black open field arena (50 cm × 50 cm), enabling 4 mice to be evaluated simultaneously [21]. After a 30-min acclimatization in the test room, 4 mice were placed simultaneously in the center of the arena and allowed to explore freely. The duration each mouse spent in the central zone was recorded using EthoVision 7.0 software (Noldus, Wageningen, The Netherlands). Longer central zone duration indicated lower anxiety, while increased peripheral activity reflected greater anxiety [22,23].

#### 2.3.2. Tail Suspension Test (TST)

Mice were suspended via tape from a metal rod 30 cm above the ground for 5 min, with tape secured 1–2 cm from the tail tip. Three mice were tested simultaneously, separated by black partitions, hand immobility time was recorded using EthoVision 7.0 software (Noldus, Wageningen, The Netherlands). Immobility was defined as the absence of struggling or pendulum-like movements, accompanied by huddling and inactivity—behaviors indicative of depressive-like states. Immobility duration correlates with the severity of depressive-like symptoms [24,25].

#### 2.3.3. Rotarod Test (RT)

Motor coordination and fatigue were evaluated using a mouse-specific rotarod (Long Bio, Shanghai, China). Mice were acclimatized for at least 5 min before testing. Following a 30-min rest, mice were initially placed on the rotarod for 10 s; recording commenced once gait and balance were stabilized. Rotor speed accelerated from 0 to 4 rpm over 10 s, then from 4 to 40 rpm over 5 min, until mice fell [26]. Latency to fall was recorded. Shorter time on the rotarod indicated greater fatigue [27].

All behavioral testing equipment must be spaced sufficiently apart to ensure independent and accurate behavioral recording for each mouse. Experimental mice in each cage were randomly grouped and sequenced prior to testing to prevent bias arising from differences in testing order or time periods between groups. After each experimental session, all testing areas and partitions were thoroughly cleaned with 75% ethanol, followed by secondary cleaning with deionized water [28]. The equipment was allowed to dry completely before the next round of testing to eliminate potential residual odor effects on subsequent animals.

### 2.4. Histopathological Examination

Mouse liver tissues were fixed in 4% paraformaldehyde until histopathological analysis. Tissues were then paraffin-embedded, sectioned at 5 μm thickness, and stained with H&E or Sirius red. Lipid accumulation and fibrosis were evaluated following standard protocols. According to the NASH-CRN system, the NASH CRN activity score (NAS) and fibrosis were independently assessed by two pathologists using a blinded approach. NAS is a semi-quantitative indicator of disease activity rather than a diagnostic classification. It comprises steatosis (0–3 points), lobular inflammation (0–3 points), and ballooning degeneration (0–2 points), with a total score range of 0–8 points (scores 0–2: non-NASH; 3–4: borderline NASH; 5–8: NASH) [29]. For Oil Red O (ORO) staining, frozen liver tissues were embedded in optimal cutting temperature (OCT) compound, sectioned at 5 μm thickness, and stained. Stained images were captured using a microscope (Leica, Wetzlar, Germany). Semi-quantitative analysis of lipid droplet size (ORO staining) and fibrotic areas (Sirius red staining) was performed using ImageJ (version 1.50k).

### 2.5. Detection of Biochemical Indicators

Serum alanine transaminase (ALT), aspartate transaminase (AST), liver triglycerides (TG), and total cholesterol (TC) were measured using commercial assay kits (Jiancheng Bioengineering Institute, Nanjing, China). The intraperitoneal glucose tolerance test (IPGTT) was performed after a 12-h fast. Animals were administered glucose (2 mg/g of body weight) dissolved in sterile water via intraperitoneal injection. Tail vein blood glucose levels were measured at 0, 30, 60, 90, and 120 min after glucose injection. Materials used in these experiments are detailed in Table S1.

### 2.6. Real-Time Quantitative Fluorescent Polymerase Chain Reaction (qRT-PCR) Analysis

To determine the mRNA expression of IL-6, IL-1β, TNF-α, and IL-18 in mouse liver, total RNA was extracted from liver tissues using the VeZol-Pure Total RNA Isolation Kit (RC202-01, VAZYME, San Diego, CA, USA). Complementary DNA (cDNA) was synthesized via reverse transcription using HiScript^®®^ II Q RT SuperMix for qPCR (+gDNA wiper) (R223-01, VAZYME, San Diego, CA, USA). Gene expression analysis was conducted through quantitative real-time PCR using the VAZYME Taq Pro Universal SYBR qPCR Master Mix (Q712-02) (San Diego, CA, USA) on a Bio-Rad CFX Connect Real-Time PCR Detection System (Hercules, CA, USA). The thermal cycling protocol consisted of an initial denaturation at 95 °C for 30 s, followed by 40 cycles of 95 °C for 3 s and 60 °C for 10 s. A melting curve analysis was subsequently performed. The 2^−ΔΔCt^ method was used to determine relative expression levels, with GAPDH serving as the endogenous control for normalization. Primer sequences are provided in Table S2.

### 2.7. High-Throughput 16S Ribosomal RNA Gene Sequencing and Analysis

Before anesthesia and blood collection, fresh feces (>1 g) from each mouse were collected, immediately frozen in 1.5 mL tubes, placed in liquid nitrogen, and stored at −80 °C until analysis. Total DNA was extracted using HiPure Stool DNA Kits (D3141, Meiji Bio, Guangzhou, China), followed by amplification and purification of the hypervariable V3-V4 region of the 16S rRNA gene. Primers used were: forward (5′–3′): CCTACGGGGNGGCWGCAG; reverse (806R, 5′–3′): GGACTACHVGGGTATCTAAT. After purification of the second-round amplicons with AMPure XP magnetic beads (Beckman, Brea, CA, USA) and quantification via Qubit 3.0, libraries were constructed using the Illumina DNA Prep Kit (20060059, Illumina, San Diego, CA, USA). Library quality was assessed using the ABI StepOnePlus Real-Time PCR System (Life Technologies, Foster City, CA, USA); only qualified samples were sequenced in PE250 mode on a Novaseq 6000 (Illumina, San Diego, CA, USA). All detection and sequencing procedures were contracted to GENEDENOVO Biotechnology Company (Guangzhou, China) for completion. Raw data were filtered, spliced, clustered, and chimera-removed to obtain operational taxonomic unit (OTU) representative sequences. Sequences were taxonomically annotated to determine species relative abundances. α-diversity indices (Chao1, Shannon, Simpson) were calculated. β-diversity analyses (e.g., PCoA, PLS-DA) were performed using Unweighted_unifrac distances. KEGG metabolic pathways were predicted via PICRUSt2 to characterize the structural and functional profiles of the fecal microbiota [30,31,32].

### 2.8. Untargeted Metabolomics

An appropriate amount of mouse liver tissue was rinsed with saline and stored at −80 °C. Quality control (QC) samples were prepared via homogenization and lysis at GENEDENOVO Biotechnology Company (Guangzhou, China). Samples were separated using an Agilent 1290 Infinity ultra-high-performance liquid chromatography (UHPLC) system (Agilent Technologies, Santa Clara, CA, USA) equipped with a Waters ACQUITY UPLC BEH Amide column (Waters Corporation, Milford, MA, USA). (1.7 μm, 2.1 mm × 100 mm). QC samples were inserted into the sample queue to verify the reliability of experimental data. The detection of metabolites was carried out with an AB SCIEX Triple TOF 6600 mass spectrometer (AB Sciex Pte. Ltd., Framingham, MA, USA), which collected data in both positive and negative ionization polarities. Raw data were format-converted and processed using XCMS (v3.7.1) for peak matching against mzCloud (https://www.mzcloud.org/), mzvault, and mass list databases to achieve accurate qualitative and relative quantitative results. Data were analyzed using multivariate statistical methods (e.g., PCA, OPLS-DA). After validating model reliability via permutation tests, differential metabolites were screened based on OPLS-DA VIP values and univariate *t*-test *p*-values, followed by correlation and clustering heatmap analyses [33]. Finally, differential metabolites were subjected to KEGG annotation and enrichment analysis, with multiple-testing correction applied to identify significantly enriched pathways [34].

### 2.9. Statistical Analysis

Beyond microbiome and metabolome analysis, we employed Mantel tests and Spearman correlation analyses to evaluate associations among gut microbiota, hepatic metabolites, biochemical markers, and proinflammatory factor expression across the three groups. For variables that could not be normalized to a standard distribution via log transformation, we used the Wilcoxon signed-rank test for comparisons between two groups. For comparisons involving more than two groups, we selected univariate or multivariate analysis of variance (ANOVA), Brown–Forsythe and Welch ANOVA, or Kruskal–Wallis test (nonparametric methods) based on data normality and homogeneity of variance, followed by Dunnett’s multiple comparison test. Normality was assessed using the Shapiro–Wilk test, and homogeneity of variance was evaluated using the Brown–Forsythe test. Data are presented as mean ± standard deviation (SD). The significance level was set at 0.05; *p* < 0.05 was considered statistically significant. Based on the effect size recommendation method proposed by Tomczak M et al. [35] in 2014, the effect sizes for the primary comparisons were calculated (total effect size η^2^ and η^2^*_H_*, pairwise comparison effect sizes Hedge’s *g* and *r* values). All statistical analyses and graphing were performed using GraphPad Prism 8.0 (San Diego, CA, USA).

## 3. Results

### 3.1. Monosaccharide Composition Characteristics of PCP

Monosaccharide composition analysis of *Poria cocos polysaccharide* (PCP) revealed its primary chemical constituents. Fourier Transform Infrared Spectroscopy (FTIR) analysis identified characteristic peaks for carbohydrates: a C-O stretching absorption peak at 1020.52 cm^−1^; a C-H stretching absorption peak at 2929.12 cm^−1^; and an O-H stretching absorption peak at 3275.36 cm^−1^ [36,37]. These results further confirm the polysaccharide nature of PCP. Compositional analysis revealed that glucose (64.1%) constitutes the primary monosaccharide component of PCP, with galactose, mannose, and fucose contributing 15.38%, 14.75%, and 5.77%, respectively. These data indicate that glucose is the predominant monosaccharide in PCP, accounting for the vast majority of the total monosaccharide composition. Detailed test results are presented in Figures S1–S3.

### 3.2. PCP Ameliorates Weight Gain and Glucose Tolerance Abnormalities in NASH Mice

**Figure 1:**
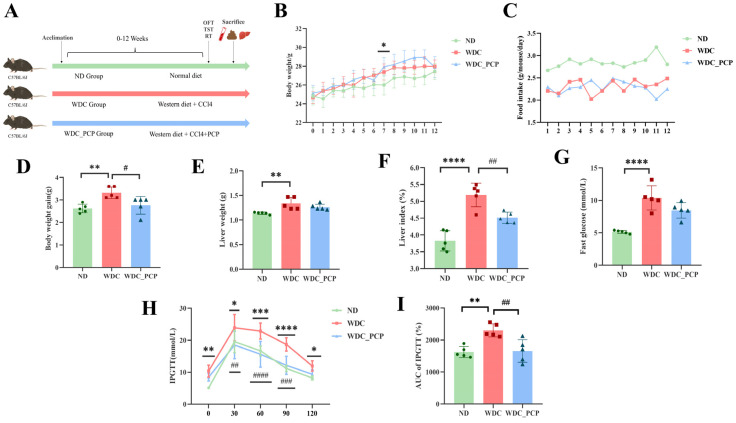
Effect of PCP on obesity and blood glucose levels in NASH mice. (**A**) Animal experimental schedule. (**B**) Weekly body weight of mice. * Indicates statistically significant differences in body weight between the ND and WDC groups, as determined by two-way ANOVA with Dunnett’s multiple comparisons test. (**C**) Food intake was recorded per cage and expressed as g/mouse/day. Data are presented as mean values from one cage (*n* = 1 cage, 5 mice) per group, and no inferential statistics were performed. (**D**) Body weight gain. (**E**) Liver weight. (**F**) Liver index (ratio of liver weight to body weight). (**G**) Fasting blood glucose. (**H**) IPGTT. *p*-values were determined using two-way ANOVA with Dunnett’s multiple comparisons test. (**I**) AUC (area under the curve of IPGTT). Normality was assessed using the Shapiro–Wilk test, and homogeneity of variance was assessed using the Brown–Forsythe test. Data are presented as mean ± SD (*n* = 5). * *p* < 0.05, ** *p* < 0.01, *** *p* < 0.001, **** *p* < 0.0001 (ND vs. WDC); # *p* < 0.05, ## *p* < 0.01, ### *p* < 0.001, #### *p* < 0.0001 (WDC vs. WDC_PCP).

To evaluate PCP’s effects on NASH, a NASH mouse model was established using a Western diet combined with intraperitoneal injections of trace amounts of CCL_4_ (Figure 1A) [16]. After 12 weeks, mice developed NASH-related phenotypes, including increased body weight, liver weight, and fasting blood glucose levels. Weight gain curves showed that from week 4 onward, WDC mice gained significantly more weight than ND mice, with a statistical difference at week 7 (*p* = 0.0257, two-way ANOVA) (Figure 1B). Throughout the study, WDC mice had significantly lower food intake than ND mice (Figure 1C) but higher weight gain (*p* = 0.0039, Kruskal–Wallis test). In contrast, weight gain in the WDC_PCP group was comparable to that in the ND group (Figure 1D). Consistent with previous reports that CCl_4_ treatment reduces food intake and mitigates Western diet-induced weight gain [16], our results showed similar trends. Liver weight was significantly increased in the WDC group (*p* < 0.01, ordinary one-way ANOVA) and reduced after PCP treatment (Figure 1E). The liver weight/body weight ratio followed a similar pattern (ND vs. WDC: *p* < 0.0001; WDC vs. WDC_PCP: *p* = 0.005) (Figure 1F). At week 12, fasting blood glucose levels and IPGTT were performed to assess glucose metabolism. Fasting blood glucose (FG) in WDC mice was approximately twice that in ND mice (*p* < 0.0001, ordinary one-way ANOVA) (Figure 1G). Glucose levels peaked at 30 min post-injection in all groups, followed by a gradual decrease. Compared to ND mice, WDC mice maintained elevated blood glucose levels at 60, 90, and 120 min (Figure 1H). The calculation of the area under the curve (AUC) for the intraperitoneal glucose tolerance test (IPGTT) demonstrated statistically significant variations when comparing the WDC cohort to both the ND and WDC_PCP groups. (ND vs. WDC: *p* = 0.0024; WDC vs. WDC_PCP: *p* = 0.0034, ordinary one-way ANOVA) (Figure 1I). These findings indicate that PCP has therapeutic potential in improving obesity and glucose tolerance in NASH mice.

### 3.3. PCP Alleviates Hepatic Lipid Deposition and Liver Injury in NASH Mice

**Figure 2:**
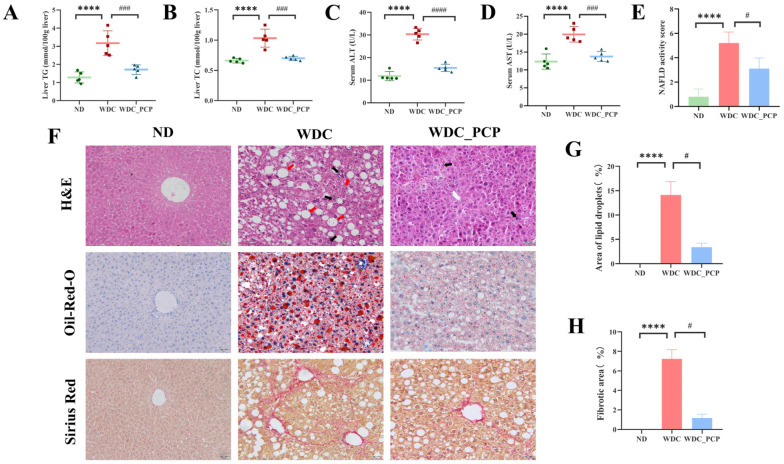
PCP ameliorates liver injury, lipid accumulation, inflammation, and fibrosis in NASH mice. (**A**) Hepatic TG. (**B**) Hepatic TC. (**C**) Serum ALT levels. (**D**) Serum AST levels. (**E**) NAFLD activity score, reflecting hepatic lobular steatosis, inflammatory infiltration, and hepatocellular ballooning. (**F**) Representative images of H&E, Oil Red O, and Sirius Red staining of liver tissues from the ND, WDC, and WDC_PCP groups (original magnification ×200; scale bar = 50 μm). Red arrows indicate steatosis; black arrows indicate inflammatory infiltration. (**G**) Semi-quantitative analysis of lipid droplets in Oil Red O-stained fields. (**H**) Semi-quantitative analysis of Sirius Red-positive hepatic areas, reflecting hepatic fibrosis severity. Normality was assessed using the Shapiro–Wilk test, and homogeneity of variance was evaluated using the Brown–Forsythe test. Histological semi-quantitative data are presented as mean ± SD, with *n* = 3 mice per group. Other data are presented as mean ± SD (*n* = 5). **** *p* < 0.0001 (ND vs. WDC); # *p* < 0.05, ### *p* < 0.001, #### *p* < 0.0001 (WDC vs. WDC_PCP). *p*-values were determined by the Kruskal–Wallis test followed by Dunnett’s multiple comparisons test or one-way ANOVA with Dunnett’s multiple comparisons test.

To assess PCP’s effects on hepatic lipid metabolism, liver function, and histopathology in NASH mice, we measured liver TG and TC levels, serum ALT and AST levels, and performed histological staining (H&E, Oil Red O, Sirius red) to evaluate lipid deposition and liver damage. Western diet combined with intraperitoneal injections of trace amounts of CCL_4_ induced elevated liver TG and TC levels, which were attenuated by PCP treatment (TG: ND vs. WDC, Hedge’s *g* = 3.55, *p* < 0.0001, 95% CI: 1.161 to 2.619; WDC vs. WDC_PCP, Hedge’s *g* = 2.79, *p* = 0.0006, 95% CI: 0.7268 to 2.185, η^2^ = 0.794; TC: ND vs. WDC, Hedge’s *g* = 3.45, *p* < 0.0001, 95% CI: 0.2291 to 0.5149; WDC vs. WDC_PCP, Hedge’s *g* = 3.11, *p* = 0.0001, 95% CI: 0.1911 to 0.4769, η^2^ = 0.811; all ordinary one-way ANOVA) (Figure 2A,B). Serum ALT and AST levels were significantly higher in the WDC group than in the ND group, with ALT levels nearly threefold elevated (ND = 11.82 ± 1.981 vs. WDC = 30.36 ± 2.580). PCP treatment reduced these levels (ALT: ND vs. WDC, Hedge’s *g* = 8.06, *p* < 0.0001, 95% CI: 15.20 to 21.87; WDC vs. WDC_PCP, Hedge’s *g* = 6.89, *p* < 0.0001, 95% CI: 11.58 to 18.25, η^2^ = 0.948; AST: ND vs. WDC, Hedge’s *g* = 3.53, *p* < 0.0001, 95% CI: 4.538 to 10.65; WDC vs. WDC_PCP, Hedge’s *g* = 3.40, *p* = 0.0005, 95% CI: 3.120 to 9.228, η^2^ = 0.785; all ordinary one-way ANOVA) (Figure 2C,D). Histologically, the Western diet combined with intraperitoneal injections of trace amounts of CCL_4_ induced NASH phenotypes, including steatosis, lobular inflammation, hepatocyte ballooning, and fibrosis—evident as large lipid droplets (Oil Red O), lipid vacuoles with swollen hepatocytes (H&E), and lobular-connected fibrosis (Sirius red) (Figure 2F). Compared to the WDC group, the WDC_PCP group showed fewer lipid vacuoles, reduced fibrosis, and a lower NASH CRN activity score (NAS) (ND = 0.80 ± 0.633 vs. WDC = 5.20 ± 0.919: *r* =1.57, *p* < 0.0001; WDC vs. WDC_PCP = 3.10 ± 0.876: *r* = 0.74, *p* = 0.0402; η^2^*_H_* = 0.837, Kruskal–Wallis test) (Figure 2E). Semi-quantitative confirmed these findings: Oil Red O staining showed hepatic steatosis rate reached 15% in the WDC group, which was reduced by 10% with PCP (ND = 0 vs. WDC = 14.12 ± 2.700: *r* = 1.16, *p* < 0.0001; WDC vs. WDC_PCP = 3.37 ± 0.855: *r* = 0.58, *p* = 0.0131; η^2^*_H_* = 0.919, Kruskal–Wallis test) (Figure 2G). Sirius red staining demonstrated PCP significantly decreased fibrotic area and collagen deposition in WDC mice (ND = 0 vs. WDC = 7.23 ± 0.930: *r* = 1.16, *p* < 0.0001; WDC vs. WDC_PCP = 1.17 ± 0.381: *r* = 0.58, *p* = 0.0131; η^2^*_H_* = 0.919, Kruskal–Wallis test) (Figure 2H). These results indicate that PCP attenuates hepatic lipid deposition and mitigates liver injury in NASH mice.

### 3.4. PCP Reduces the Expression of Pro-Inflammatory Factors in the Liver of NASH Mice

**Figure 3:**
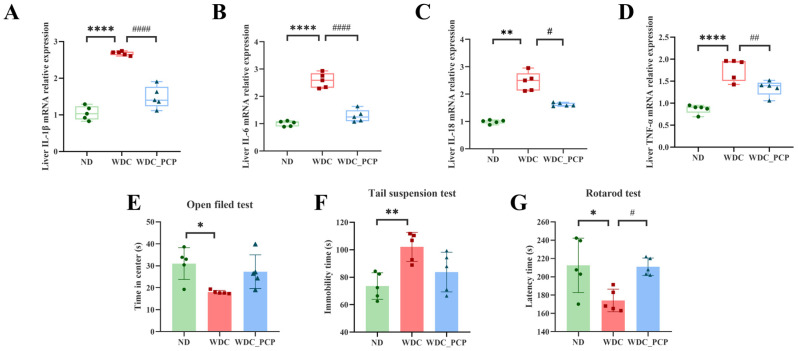
PCP decreases the expression of pro-inflammatory factors and ameliorates fatigue-like performance on the rotarod test in NASH mice. (**A**–**D**) Relative mRNA expression levels of pro-inflammatory factors IL-1β, IL-6, IL-18, and TNF-α in the liver. (**E**) Time spent in the central region by mice in the OFT over 5 min. (**F**) The immobility time of mice in the TST was within 5 min. (**G**) Time mice remained on the rotarod in the RT. Normality was assessed using the Shapiro–Wilk test, and homogeneity of variance was evaluated using the Brown–Forsythe test. Data are presented as mean ± SD (*n* = 5). * *p* < 0.05, ** *p* < 0.01, **** *p* < 0.0001 (ND vs. WDC); # *p* < 0.05, ## *p* < 0.01, #### *p* < 0.0001 (WDC vs. WDC_PCP). *p*-values were determined by one-way ANOVA with Dunnett’s multiple comparisons test.

To evaluate changes in hepatic pro-inflammatory factor mRNA expression, we performed RT-qPCR to measure the relative mRNA levels of IL-1β, IL-6, IL-18, and TNF-α across the three groups. Results showed significantly lower mRNA expression of IL-1β, IL-6, IL-18, and TNF-α in liver tissues of the WDC_PCP group compared to the WDC group (IL-1β and IL-6: *p* < 0.0001, ordinary one-way ANOVA; IL-18: *p* = 0.0106, Brown–Forsythe and Welch ANOVA; TNF-α: *p* = 0.0061, ordinary one-way ANOVA) (Figure 3A–D). These findings indicate that PCP may slow NASH progression by reducing pro-inflammatory factor mRNA expression.

### 3.5. PCP Alleviates Fatigue-like Performance on Rotarod in NASH Mice

Evidence indicates that high-cholesterol diet-induced obese mice exhibit anxiety- and depression-like behaviors and increased fatigability [19]. In the present study, we conducted a series of behavioral tests to investigate whether such manifestations occur in NASH mice and whether PCP exerts ameliorative effects. Compared with ND mice, WDC mice showed significantly less time in the central zone during the OFT (*p* < 0.05, 95% CI: −22.64 to −3.341, ordinary one-way ANOVA) (Figure 3E), significantly longer immobility time in the TST (*p* < 0.01, 95% CI: 9.956 to 47.08, ordinary one-way ANOVA) (Figure 3F), and significantly shorter fall latency in the RT (*p* < 0.05, 95% CI: −69.00 to −7.681, ordinary one-way ANOVA) (Figure 3G). These results indicate that a Western diet combined with intraperitoneal injections of trace amounts of CCL_4_ can prolong the central dwell time and immobility time in NASH mice during the open field test and tail suspension test, while also inducing fatigue-like performance on the rotarod test. However, PCP did not significantly improve the outcomes of the open field test and tail suspension test in NASH mice, though a reversal trend was observed in both tests. Notably, PCP intervention significantly increased rotarod retention time in NASH mice (*p* < 0.05, 95% CI: −67.48 to −6.161, ordinary one-way ANOVA) (Figure 3G), indicating PCP’s potential to alleviate fatigue-like performance on the rotarod test in NASH mice.

### 3.6. PCP Improves the Structure and Composition of Gut Microbiota in NASH Mice

**Figure 4:**
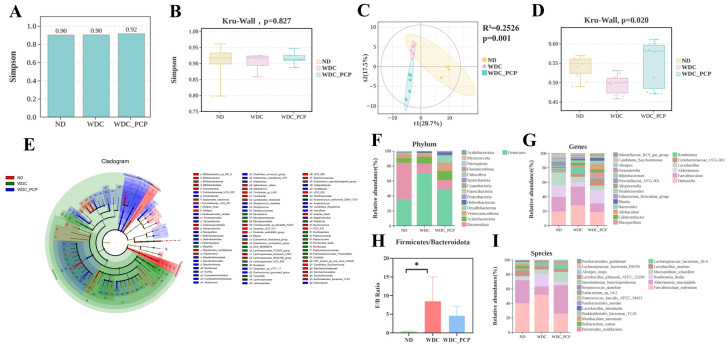
Effect of PCP on the gut microbiome in NASH mice. (**A**,**B**) Alpha diversity represented by Simpson’s index; *p*-values were determined by the Kruskal–Wallis test. (**C**) Partial least squares discrimination analysis (PLS-DA) based on unweighted_unifrac revealed a separation in fecal microbial structure among the three groups (ND: *n* = 5, yellow region; WDC: *n* = 5, red region; WDC_PCP: *n* = 5, green region). PERMANOVA analysis yielded an R^2^ value of 0.2526 and a *p*-value of 0.001, confirming statistically significant differences among groups. (**D**) Differences in fecal microbial β-diversity among the three groups, with *p*-values obtained from the Kruskal–Wallis test. (**E**) Biomarker features for each group were identified via LEfSe analysis with screening criteria of LDA score >2.00 and *p* < 0.05, yielding 98 differentially abundant microorganisms (ND vs. WDC vs. WDC_PCP). (**F**) Microbial composition at the phylum level. (**G**) Microbial composition at the genus level. (**H**) *Firmicutes*-to-*Bacteroidetes* ratio. * *p* < 0.05 (ND vs. WDC), normality was assessed using the Shapiro–Wilk test, and homogeneity of variance was evaluated using the Brown–Forsythe test. *p*-values were determined by one-way ANOVA with Dunnett’s multiple comparisons test. (**I**) Microbial composition at the species level.

Mounting evidence links NASH development to gut dysbiosis [38]. To characterize gut microbial features in NASH mice and explore how PCP exerts therapeutic effects via microbiota modulation, we performed 16S rRNA gene sequencing (V3–V4 region) on fecal samples from the three groups. α-diversity assessment using Simpson’s index showed that neither Western diet combined with intraperitoneal injections of trace amounts of CCL_4_ nor PCP treatment significantly altered gut microbial richness or evenness (Figure 4A,B). Analysis of β-diversity demonstrated clear separation in fecal microbiota composition across the three experimental groups, as determined by Unweighted_uniFrac, a phylogenetic-based metric, and partial least squares discriminant analysis (PLS-DA) (ND: *n* = 5, blue; WDC: *n* = 5, red; WDC_PCP: *n* = 5, green). Simultaneously, we performed a PERMANOVA test on the unweighted_unifrac distances among the three groups, yielding R^2^ = 0.2526, *p* = 0.001 (Figure 4C and Figure S4). Kruskal–Wallis testing confirmed significant differences in microbial structure across groups (*p* = 0.02) (Figure 4D). To clarify compositional shifts, we analyzed microbiota at the phylum, genus, and species levels. Linear discriminant analysis (LDA) coupled with LEfSe identified 98 differential taxa (filtered by LDA > 2, *p* < 0.05) [39,40] (Figure 4E). Analysis of species composition revealed that Firmicutes and Bacteroidetes, as dominant gut microbiota, exhibited a significantly increased ratio between the two in the WDC group and a significantly decreased ratio in the PCP group (Figure 4F,H). At the genus level, NASH mice (WDC group) showed significantly higher relative abundances of *Romboutsia*, *Bilophila*, *Roseburia*, *Paludicola*, *Tuzzerella*, *Lachnospiraceae_UCG-006*, and *Mycoplasma*, and lower abundances of *Alistipes*, *Harryflintia*, and *Butyricoccaceae_UCG-009* compared with ND mice. PCP treatment reversed these trends (Figure 4G). Among the three groups, *Romboutsia* and *Alistipes* were the two genera contributing most significantly. Following PCP treatment, the relative abundance of *Romboutsia* decreased by approximately 4.06 (*p* = 0.132, Kruskal–Wallis test), while the relative abundance of Alistipes increased by approximately 0.061 (*p* = 0.700, 95% CI: −0.2816 to 0.1599, Brown–Forsythe and Welch ANOVA tests). Consistent with previous findings that lychee-derived polyphenols alleviate hepatic steatosis by reducing *Romboutsia* abundance [41], *Romboutsia_ilealis* (a species within *Romboutsia*) also showed significantly higher relative abundance in the WDC group (LDA = 4.36, *p* = 0.027) (Figure 4I). These results suggest that PCP may mitigate Western diet combined with intraperitoneal injections of trace amounts of CCL_4_-induced liver injury in NASH mice by regulating gut microbiota composition.

### 3.7. PCP Improves Hepatic Untargeted Metabolite Profiles in NASH Mice

**Figure 5:**
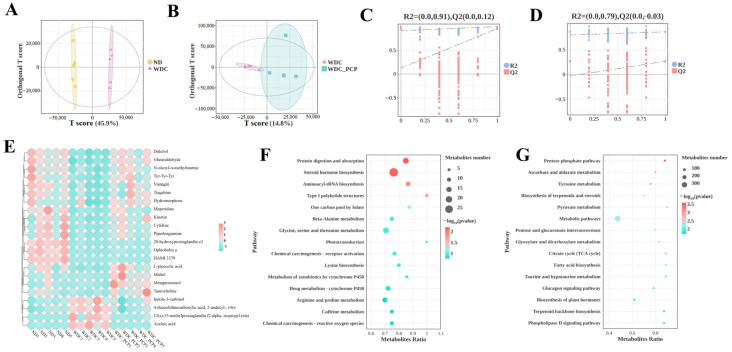
PCP alters hepatic metabolites in NASH mice. (**A**,**B**) OPLS-DA score plots for ND vs. WDC vs. WDC_PCP. (**C**,**D**) Accuracy assessment of OPLS-DA via permutation test. (**E**) Up- and down-regulation patterns of 22 differential hepatic metabolites among the three groups (ND, WDC, WDC_PCP); the legend indicates the fold change in regulation. (**F**,**G**) Top 15 significantly enriched pathways from KEGG enrichment analysis in positive and negative ion modes, with circle size representing the number of corresponding metabolites.

**Figure 6:**
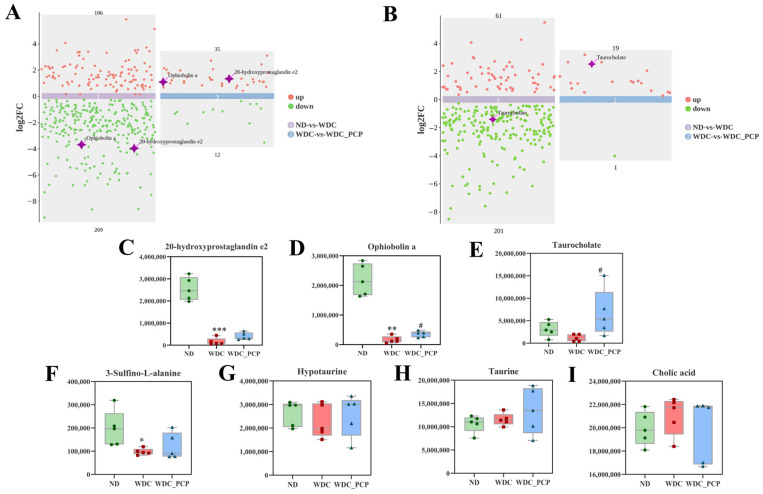
Key differential metabolites in hepatic metabolomics of NASH mice indicate potential therapeutic targets of PCP. (**A**,**B**) Regulation patterns of hepatic metabolites and key differential metabolites in each group under positive and negative ion modes (based on *p* < 0.05, VIP > 1, log_2_FC ≥ 1 or ≤−1; red dots represent up-regulated metabolites, green dots represent down-regulated metabolites, and purple stars represent key differential metabolites). (**C**–**E**) Levels of key differential metabolites 20-hydroxyprostaglandin e2, ophiobolin a, and taurocholate in the three groups. (**F**–**I**) Changes in upstream metabolites (3-Sulfino-L-alanine, hypotaurine, taurine, and cholic acid) of the key differential pathway taurine and hypotaurine metabolism. Normality was assessed using the Shapiro–Wilk test, and homogeneity of variance was evaluated using the Brown–Forsythe test. Data are presented as mean ± SD (*n* = 5). * *p* < 0.05, ** *p* < 0.01, *** *p* < 0.001 (ND vs. WDC); # *p* < 0.05 (WDC vs. WDC_PCP). *p*-values for taurocholate and cholic acid were determined by Kruskal–Wallis test with Dunnett’s multiple comparisons test; *p*-values for 20-hydroxyprostaglandin e2, ophiobolin a, and taurine were determined by Brown–Forsythe and Welch ANOVA with Dunnett’s T3 multiple comparisons test; *p*-values for 3-Sulfino-L-alanine and hypotaurine were determined by one-way ANOVA with Dunnett’s multiple comparisons test.

To investigate hepatic metabolomic characteristics and the regulatory role of PCP in hepatic metabolomics of NASH mice, we performed untargeted metabolomic analysis on liver tissues from three groups. The OPLS-DA model was established to analyze metabolic changes between groups and intra-group variations, with significant separation observed among ND, WDC, and WDC_PCP groups in OPLS-DA score plots (Figure 5A,B). Permutation test results confirmed no model overfitting (Figure 5C,D). Following quality control and normalization, differential metabolites were filtered using thresholds of *p* < 0.05, VIP > 1, and log_2_FC ≥ 1 or ≤−1. Metabolomic profiling revealed 22 significantly altered metabolites relative to the ND group, comprising 10 that were up-regulated and 12 that were down-regulated (Figure 5A). Further screening to identify key differential metabolites identified 15 significantly up- and down-regulated differential metabolites compared to the control. 3 of the key differential metabolites were of particular interest (Figure 6A,B), namely 20-hydroxyprostaglandin e2, ophiobolin a, and taurocholate, which were induced by the Western diet combined with intraperitoneal injections of trace amounts of CCL_4_, all showed significant downregulation. After PCP treatment, the ophiobolin a, and taurocholate levels were significantly upregulated (ophiobolin a: *p* = 0.0284, Brown–Forsythe and Welch ANOVA; taurocholate: *p* = 0.0178, Kruskal–Wallis test) (Figure 6C–F). Kyoto Encyclopedia of Genes and Genomes (KEGG) enrichment analysis was used to explore metabolic pathways associated with differential metabolites and understand PCP’s effects on liver metabolism in NASH mice. Significant metabolite enrichment was observed in the citrate cycle (TCA cycle), fatty acid biosynthesis, and taurine and hypotaurine metabolism (*p* < 0.05). Additionally, metabolites showed enrichment in glycine, serine, and threonine metabolism, beta-alanine metabolism, and lysine biosynthesis, though these were not statistically significant (*p* > 0.05) (Figure 5F,G). Notably, taurocholate—one of the key differential metabolites—was significantly enriched in taurine and hypotaurine metabolism (*p* = 0.008). These results suggest PCP may act on NASH mice by regulating anti-inflammatory and lipid metabolites, potentially through pathways including taurine and hypotaurine metabolism. Given that taurocholate is a downstream metabolite in taurine and hypotaurine metabolism, we further investigated pathway changes and PCP’s potential therapeutic mechanisms by examining taurocholate’s upstream metabolites (3-sulfino-L-alanine, hypotaurine, taurine, and cholic acid) across the three groups. Hypotaurine and taurine levels were comparable among groups. Compared with ND mice, WDC mice showed significantly down-regulated 3-sulfino-L-alanine (*p* = 0.032, ordinary one-way ANOVA), with a trend toward up-regulation after PCP intervention. Cholic acid levels showed an up-regulation trend followed by down-regulation (Figure 6F–I).

### 3.8. Association Between PCP-Regulated Key Differential Metabolites and Gut Microbiota

**Figure 7:**
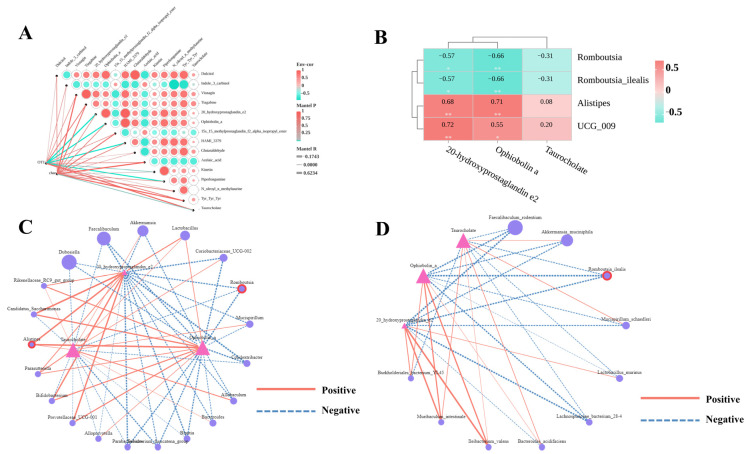
Correlation analysis of hepatic differential metabolites with the gut microbiome. (**A**) Mantel test correlation analysis between hepatic differential metabolites and the gut microbiome. *Mantel R* represents the correlation strength between differential metabolites and the gut microbiome. (**B**) Spearman correlation heatmap of key differential gut microbes vs. key differential hepatic metabolites, with the resulting *r*-value indicating correlation strength; the legend denotes this strength. * *p* < 0.05 or ** *p* < 0.01 in ND vs WDC or WDC vs WDC_PCP; *p*-value obtained by Spearman correlation analyses. (**C**) Spearman correlation network of key differential hepatic metabolites with genus-level gut microbes. (**D**) Spearman correlation network of key hepatic metabolites with species-level gut microbes. Triangles represent key differential metabolites; circles represent gut microbes, with circles outlined in red representing key differential gut microbes. Solid red lines indicate positive correlations, dashed blue lines indicate negative correlations, and line thickness reflects correlation strength in the network.

Potential associations between intestinal microbial communities and liver metabolite profiles were investigated using Mantel tests. These analyses evaluated the correlations involving 15 differentially abundant metabolites, with the outcomes displayed in correlation network heatmaps. At all taxonomic levels, the top 5 metabolites with the strongest effects on gut microbiota composition were: 20-hydroxyprostaglandin e2 (Mantel *R* ≥ 0.4, *p* < 0.01), ophiobolin a (Mantel *R* ≥ 0.4, *p* < 0.05), piperlongumine (Mantel *R* ≥ 0.4, *p* < 0.01), HAMI 3379 (Mantel *R* ≥ 0.4, *p* < 0.01). Taurocholate had the most pronounced impact on microbial diversity (Mantel *R* = 0.39, *p* = 0.04 for Chao1) (Figure 7A), consistent with the identification of key differential metabolites in metabolomic analyses. Subsequently, Spearman correlation analysis was used to identify taxa (at genus and species levels) associated with the 4 key differential metabolites, with results presented as correlation network diagrams and heatmaps (Figure 7C,D). At the genus level, *Romboutsia* relative abundance was significantly negatively correlated with 20-hydroxyprostaglandin e2 and ophiobolin a levels (−0.5 ≤ *r* ≤ −0.8, *p* < 0.05), while *Alistipes* relative abundance showed significant positive correlations with 20-hydroxyprostaglandin e2 and ophiobolin a levels (0.5 ≤ *r* ≤ 0.8, *p* < 0.01). At the species level, *Romboutsia_ilealis* relative abundance was significantly negatively correlated with 20-hydroxyprostaglandin e2, ophiobolin a levels (−0.5 ≤ *r* ≤ −0.8, *p* < 0.05) (Figure 7B). In summary, combined Mantel tests and Spearman correlation analyses identified *Romboutsia*, *Romboutsia_ilealis*, and *Alistipes* as key differential gut microbes across all OTU levels.

### 3.9. Association Between Key Differential Gut Microbes and Multidimensional Disease Phenotypes

**Figure 8:**
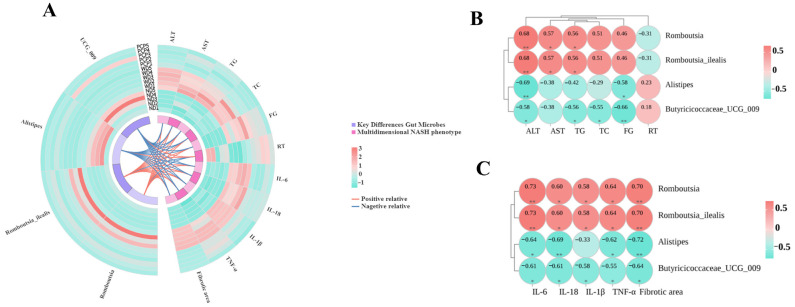
Association between key differential gut microbes and multidimensional disease phenotypes. (**A**) Circular Circos plot of Spearman correlation analysis between key differential gut microbes and multidimensional disease phenotypes. Key differential gut microbes are shown on the left; biochemical indices, pro-inflammatory factors (IL-1β, IL-6, IL-18, TNF-α), mouse fatigue-like performance on the rotarod test, and hepatic fibrosis severity are shown on the right. The outer heatmap indicates relative abundance in each sample, and the middle lines represent correlations between elements on both sides (red lines: positive correlations; blue lines: negative correlations). (**B**) Spearman correlation heatmap of key differential gut microbes (derived from LEfSe analysis) with biochemical and behavioral metrics. (**C**) Spearman correlation heatmap of key differential gut microbes (derived from LEfSe analysis) with pro-inflammatory factors and hepatic fibrosis severity. Spearman correlation *r*-values indicate correlation strength. Red denotes positive correlations; green denotes negative correlations. * *p* < 0.05 or ** *p* < 0.01 in ND vs. WDC or WDC vs. WDC_PCP; *p*-value obtained by Spearman correlation analyses.

Spearman correlation analysis was used to generate dynamic Circos plots and correlation heatmaps (Figure 8A) to explore further associations between key differential gut microbes and biochemical indices, behavioral parameters, and pro-inflammatory factor levels. Results showed that the relative abundance of *Romboutsia* and *Romboutsia_ilealis* was significantly positively correlated with ALT, AST, and TG levels. In contrast, *Alistipes* abundance was significantly negatively correlated with ALT and FG levels. The relative abundance of *Butyricoccaceae_UCG-009* was significantly negatively correlated with ALT, TG, TC, and FG levels. All four gut microbes showed moderate correlations with ALT levels (0.5 < |*r|* < 0.8, *p* < 0.05) (Figure 8B). Analysis of behavioral parameters revealed a positive association between the relative abundance of *Alistipes* and *Butyricoccaceae_UCG-009* with RT performance. Conversely, an inverse relationship was observed for *Romboutsia* and *Romboutsia_ilealis*. Regarding systemic inflammation, the abundances of *Romboutsia* and *Romboutsia_ilealis* demonstrated significant positive correlations with the expression levels of pro-inflammatory mediators, including IL-6, IL-1β, IL-18, and TNF-α. In contrast, *Alistipes* and *Butyricoccaceae_UCG-009* showed significant negative correlations with these inflammatory cytokines, with most associations being of moderate strength (Figure 8C).

## 4. Discussion

In the pathological progression of NASH, hepatocytes exhibit abnormal lipid accumulation, excessive oxidative stress, and persistent inflammation. These factors synergize to damage hepatic parenchymal cells. Simultaneously, activation of innate immune cells within the liver leads to the release of pro-inflammatory factors. The establishment of an inflammatory microenvironment activates the transformation of hepatic stellate cells into myofibroblasts and triggers massive synthesis of extracellular matrix, driving the onset and progression of hepatic fibrosis [42]. Thus, current therapeutic strategies for NASH focus on alleviating metabolic dysregulation and cellular injury, while directly targeting subsequent inflammation and fibrosis [43]. Conversely, growing evidence highlights a close link between intestinal dysbiosis and the progression of NAFLD, NASH, and even NAFLD-associated HCC [44]. The intestine and liver are closely connected via the portal vein, and coupled with their shared embryonic origin, the gut–liver axis plays a pivotal role in regulating metabolic diseases and maintaining metabolic homeostasis [45]. Studies have shown that natural phytopolysaccharides extracted from traditional Chinese medicine formulations exert preventive and therapeutic effects on complex conditions such as malignant tumors and metabolic diseases, with multifaceted pharmacological activities including immunomodulation, antioxidant, anti-inflammatory, and antitumor properties [46,47]. Additionally, they can regulate intestinal flora to influence therapeutic outcomes [48,49,50]. As demonstrated by Yi-yun Tan et al. [51], PCP can regulate the NF-κB/CCL3/CCR1 axis, thereby reducing hepatic injury and inflammation in NASH mice. However, the specific mechanism underlying PCP’s efficacy—particularly its interplay with intestinal flora and metabolomics—remains unclear. In this study, we extracted and analyzed the components of *Poria cocos polysaccharides*, finding that PCP alleviated pathological manifestations in NASH mice induced by a Western diet combined with intraperitoneal injections of trace amounts of CCL_4_. Using 16S rRNA gene sequencing, we analyzed PCP’s effects on the composition and diversity of intestinal microbiota in NASH mice. We further investigated PCP’s effects on NASH mouse livers via untargeted metabolomics, analyzed metabolites, and explored their associations with key gut microbes to clarify the underlying mechanism.

The therapeutic effects of *Poria cocos* on liver diseases are reflected in changes in intestinal flora, hepatic metabolites, and liver expression of pro-inflammatory factors [52,53]. As the main active component of *Poria cocos*, PCP has demonstrated anti-inflammatory, antitumor, and antioxidant activities [53,54]. Numerous studies have shown that PCP can reduce inflammation and lipid levels to alleviate atherosclerosis [55] and ameliorate acetaminophen-induced liver injury [56]. These findings support the potential application of PCP in treating liver diseases. In this study, we administered PCP to mice at a dose of 236 mg/kg/day. This dosage was selected based on the dose range used in similar studies in the literature, which demonstrated positive effects on NASH and fatigue-like performance without causing significant toxicity or side effects. In the present study, we confirmed that a Western diet combined with intraperitoneal injections of trace amounts of CCL_4_ successfully induced NASH, characterized by weight gain and increased hepatic fat; however, PCP did not significantly improve body weight. Notably, PCP effectively regulated hepatic lipid metabolism and reduced fat accumulation, as evidenced by changes in liver-to-body weight ratio and hepatic lipid levels, providing strong evidence for its therapeutic efficacy. Furthermore, observations of IPGTT changes and hepatic AST/ALT levels indicated that PCP improved glucose metabolism and mitigated liver injury.

Growing evidence links gut microbiota disruption to NAFLD progression. As NASH advances, improving gut microbial composition may emerge as a key therapeutic strategy [57]. PCPs’ anti-inflammatory properties and ability to modulate gut microbiota suggest they could ameliorate NASH [58]. To investigate this premise, we conducted 16S rRNA gene sequencing on murine fecal samples. The analysis indicated that a Western diet induces gut microbiota dysbiosis, marked by diminished microbial diversity, reduction in beneficial bacterial taxa, and expansion of pathogenic bacterial populations. The PCP restored the intestinal flora structure toward a normal state. Based on LEfSe analysis, we identified 3 key discriminant bacteria that are highly correlated with NASH progression and selectively regulated by PCP. Although other microbial changes were observed, these three bacteria made the most significant contribution, representing more robust microbial markers of PCP-mediated NASH improvement. This aligns with the notion that key bacteria drive microbiota–host interactions in NASH [51,59]. At both the genus and species levels, NASH mice showed significant enrichment of potentially pathogenic *Romboutsia* and *Romboutsia ilealis*. While few studies have directly linked *Romboutsia_ilealis* to NASH, it is generally recognized as a “pathogenic commensal,” consistent with its anaerobic lifestyle [50,60]. In obese adolescents, *Romboutsia_ilealis* is significantly enriched in NAFLD patients and participates in pyruvate fermentation to produce lactate and acetate—the least anti-inflammatory SCFAs. This suggests its increased relative abundance strongly associates with hepatic inflammation and metabolic disturbances [49,50,61,62]. In our study, PCP intervention significantly downregulated *Romboutsia_ilealis* abundance in NASH mice, consistent with existing research. This therapeutic effect may involve maintaining intestinal flora homeostasis and disrupting pyruvate metabolism to enhance anti-inflammatory capacity, ultimately exerting a beneficial effect on NASH. In NASH mouse feces, *Alistipes* was significantly downregulated, and this reduction was reversed by PCP—consistent with findings that various plant polysaccharides can restore *Alistipes* levels. For example, sulfated polysaccharides from *Gracilaria lemaneiformis* modulate cholesterol and bile acid metabolism in high-fat diet-fed mice [63], and *Grifola frondosa* heteropolysaccharides attenuate inflammatory damage in NAFLD rats, similar to the reported effects of *Alistipes* [64]. In the context of liver disease progression, *Alistipes* exhibits hepatoprotective effects, and reduced abundance correlates with liver fibrosis [65]. More broadly, *Alistipes* is a unique microbial genus associated with aging phenotypes; it is bile-resistant, undergoes distinctive amino acid fermentation to synthesize lysine, and produces SCFAs [66], earning it recognition as a “potential probiotic.” Additionally, studies indicate *Alistipes* has anti-fatigue properties, possibly linked to amino acid and central carbohydrate metabolism [67,68]. Our study showed PCP intervention significantly improved fatigue-like performance on the rotarod test in NASH mice, suggesting that increasing *Alistipes* abundance—thereby exerting anti-hepatic fibrosis, bile acid metabolism-regulating, and anti-fatigue effects—may be a key target of PCP in NASH treatment. *Butyricicoccaceae_UCG-009*, a genus in the *Butyricicoccaceae* family, showed significant negative correlations with hepatic pro-inflammatory factors, hepatic lipid levels, and liver function indices in our study. Previous research identifies *Butyricicoccaceae_UCG-009* as an SCFA-producing microbe involved in bile acid metabolism; its butyrate production inhibits high-fat diet-induced systemic inflammation, delays lipogenesis, and maintains energy homeostasis [69,70]. Thus, the significant upregulation of *Butyricicoccaceae_UCG-009* in the PCP intervention group suggests its role in PCP’s ameliorative effects on NASH.

The pathogenesis of NASH is linked to disturbances in bile acid metabolism [71]. This study revealed through non-targeted metabolomics that PCP significantly reversed the declining trend of 3-Sulfino-L-alanine, an upstream metabolite in the taurocholate and taurine synthesis pathway, in the livers of NASH mice. Concurrently, taurine levels remained stable while cholic acid levels increased. As a conjugated bile acid, taurocholate plays a crucial role in promoting lipid emulsification and digestion. Its decreased levels may exacerbate lipid accumulation and hepatocyte injury in NASH [72]. As an intermediate in the taurine synthesis pathway, the reduction in 3-sulfino-L-alanine may reflect impaired taurine synthesis capacity in the NASH state. The rebound of this metabolite following PCP intervention suggests it may exert a protective effect against NASH by restoring taurine synthesis capacity and improving conjugated bile acid metabolism. Although taurine levels did not show a synchronous decrease, this phenomenon may be related to the body maintaining taurine pool stability through other compensatory pathways, warranting further exploration in future studies. Overall, the regulatory effect of PCP on the taurocholate metabolic network may represent one of its key mechanisms for improving NASH.

Other key differential metabolites also exert anti-NASH effects. In this study, NASH mice exhibited an upward trend in ophiobolin a levels following PCP treatment. It has antitumor activity, with studies demonstrating cytotoxicity against human hepatocellular carcinoma cells (Bel-7402)—a key role in slowing disease progression [73]. This study also found significantly reduced 20-hydroxyprostaglandin e2 levels in NASH mice, with a trend toward normalization after PCP treatment. However, its potential anti-hepatic fibrosis and anti-inflammatory effects remain unclear [74]. Further research is needed to clarify how PCP regulates 20-hydroxyprostaglandin e2 levels to exert therapeutic effects, including its bidirectional regulatory mechanisms.

In this study, after PCP intervention, hepatic metabolite levels in NASH mice showed distinct trends: Taurocholate trended toward normalization, while 20-hydroxyprostaglandin e2 andophiobolin a trended toward recovery. Additionally, PCP intervention ameliorated hepatic inflammatory factor expression and reduced lipid levels in NASH mice. Correlation analyses showed that the relative abundance of the potentially pathogenic *Romboutsia_ilealis* in the gut microbiota of PCP-treated NASH mice decreased significantly, with a positive correlation between *Romboutsia_ilealis* and both inflammatory factors and hepatic lipases. Simultaneously, the relative abundance of *Alistipes* and *Butyricoccaceae_UCG-009*—taxa negatively correlated with these indices—increased significantly. Notably, their abundance showed an opposing trend to RT performance. These findings support the hypothesis that PCP may ameliorate NASH and its associated fatigue by regulating hepatic metabolism (particularly taurine and hypotaurine metabolism), likely linked to changes in the abundance of *Romboutsia_ilealis*, *Alistipes*, and *Butyricoccaceae_UCG-009*.

We must acknowledge that the NIH gender balance policy mandates equal representation of male and female animals in experimental designs [75]. The use of male mice in this study was primarily driven by methodological considerations: the estrous cycle in female mice (approximately 3–5 weeks) may cause significant fluctuations in metabolism and microbiota, potentially interfering with effect assessment [76,77]. From a clinical perspective, female NASH patients bear a heavier disease burden: epidemiological data indicate faster liver fibrosis progression and higher mortality rates among female NASH patients, with a particularly marked increase in risk post-menopause [78]. The core mechanism involves the loss of estrogenic protection [79]. Evidence-based studies have demonstrated that plant polysaccharides improve metabolic markers in postmenopausal women [80]. Notably, the key bacterium Alistipes identified in this study exhibits higher baseline abundance in the female gut and greater responsiveness to prebiotic intervention [81]. Although this study did not directly involve female animals, the potential therapeutic efficacy of PCP may hold greater application value in women. In subsequent research, we will strictly adhere to the NIH gender balance policy by including female mice to comprehensively evaluate its translational potential.

This study also has certain limitations. Complete blinding was implemented only for histological assessment (the primary subjective endpoint). Due to practical constraints in animal experiments, blinding could not be applied to other phases; however, we minimized potential bias through pre-specified experimental protocols and statistical analysis plans. When performing differential analysis using LEfSe, despite filtering based on VIP values, the risk of false positives may increase due to insufficient statistical power in small sample sizes [82]. Therefore, the LEfSe analysis results in this study are primarily used for preliminary screening of potentially differentially abundant microbes. Further validation through expanded sample sizes or experimental verification is still required to confirm their reliability.

## 5. Conclusions

Our study demonstrates the therapeutic effects of PCP on NASH in mice. PCP administration led to a reduction in hepatic lipid levels and blood glucose, along with alleviation of liver inflammation and fibrosis. Additionally, PCP improved fatigue-like performance, as evidenced by enhanced performance in the rotarod test. The beneficial effects of PCP were associated with the restoration of gut microbial diversity, promoting beneficial bacteria such as *Alistipes* and *Butyricoccaceae_UCG-009*, and inhibiting harmful bacteria like *Romboutsia ilealis*. Liver metabolomics revealed that PCP normalized key metabolites, including taurocholate, which correlates with reduced inflammation and improved fatigue symptoms. Collectively, these data underscore the potential of PCP as an edible agent to mitigate NASH by modulating the gut microbiota composition and hepatic metabolic pathways, specifically taurine and hypotaurine metabolism.

## Data Availability

The raw 16S rDNA sequencing data from this study have been uploaded to the NCBI SRA database, with the project number PRJNA1367511 (link: https://www.ncbi.nlm.nih.gov/sra/PRJNA1367511, accessed on 28 November 2025). Liver metabolomics data from this study are available upon request from the corresponding author.

## Supplementary Materials

The following supporting information can be downloaded at: https://www.mdpi.com/article/10.3390/nu17233797/s1, Figure S1. Ion Chromatography Profile of *Poria cocos* Polysaccharides (PCP) Monosaccharides; Figure S2. Standard Monosaccharide Components Ion Chromatography; Figure S3. FTIR Spectrum of *Poria cocos* Polysaccharides (PCP); Figure S4. PERMANOVA Analysis of β-Diversity Using Unweighted_uniFrac Distances. Table S1: Reagents used in the experiment; Table S2: The primer sequence of the target gene used in the study.
