# Supplementary material for: Modulation of Gut Microbes and Hepatic Metabolites by PCP Ameliorates NASH and Fatigue-like Performance in Mice
Source: Nutrients. 2025 Dec 3;17(23):3797. doi: 10.3390/nu17233797 (PMC12693755; doi:10.3390/nu17233797)
Supplement: Supplementary file 1 [file nutrients-17-03797-s001.zip › Supplementary material/Supplementary material.pdf]

Supplementary material

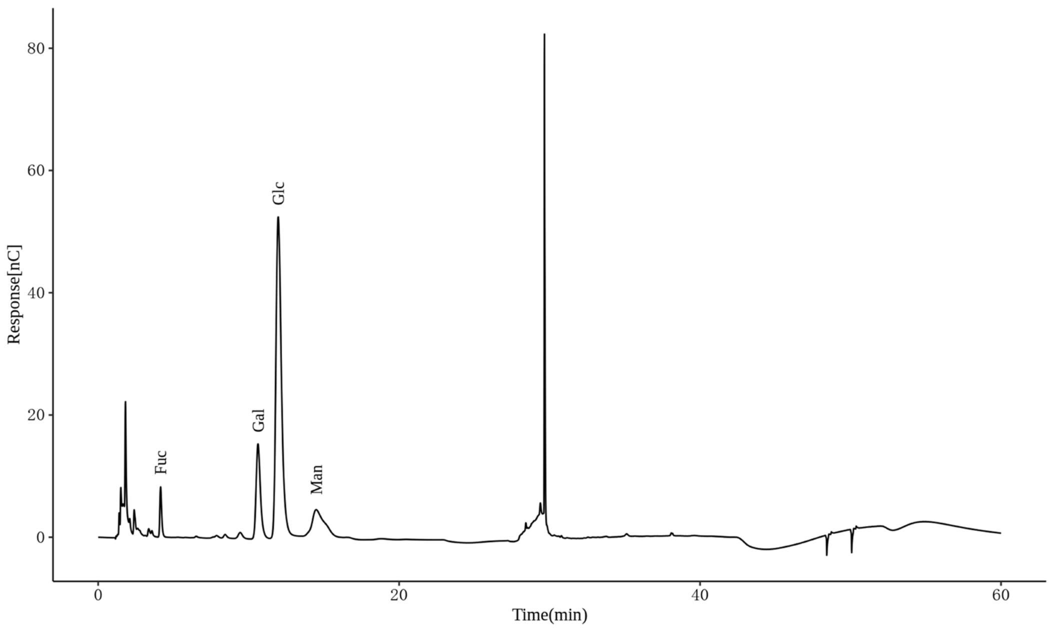

Figure S1. Ion Chromatography Profile of Poria cocos Polysaccharides (PCP) Monosaccharides. The ion chromatogram displays the separation of monosaccharides in PCP. Peaks are identified for fucose (Fuc), galactose (Gal), mannose (Man), and glucose (Glc), with glucose being the most abundant. The chromatographic conditions were optimized with a Dionex™ CarboPac™ PA20 column and a gradient elution program, providing a clear resolution of the monosaccharide components.

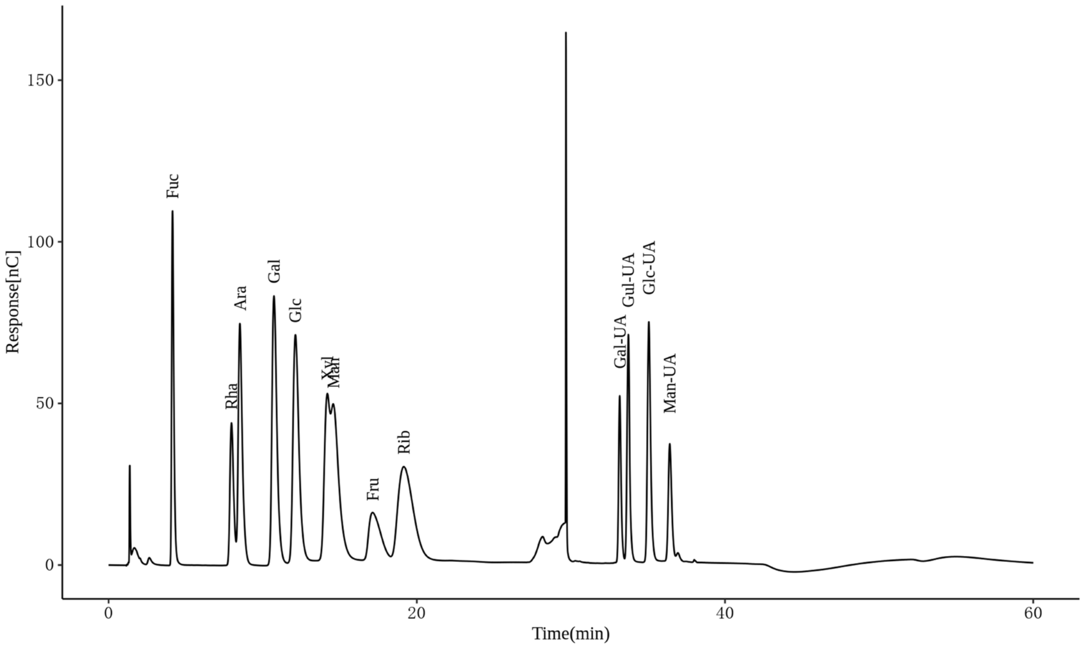

Figure S2. Standard Monosaccharide Components Ion Chromatography. This chromatogram represents the ion chromatography analysis of standard monosaccharides including fucose (Fuc), rhamnose (Rha), arabinose (Ara), galactose (Gal), glucose (Glc), xylose (Xyl), ribose (Rib), galacturonic

acid (Gal-UA), guluronic acid (Gul-UA), and mannuronic acid (Man-UA). The analysis was performed under the same conditions as Figure S1, ensuring accuracy in the identification and quantification of PCP monosaccharides.

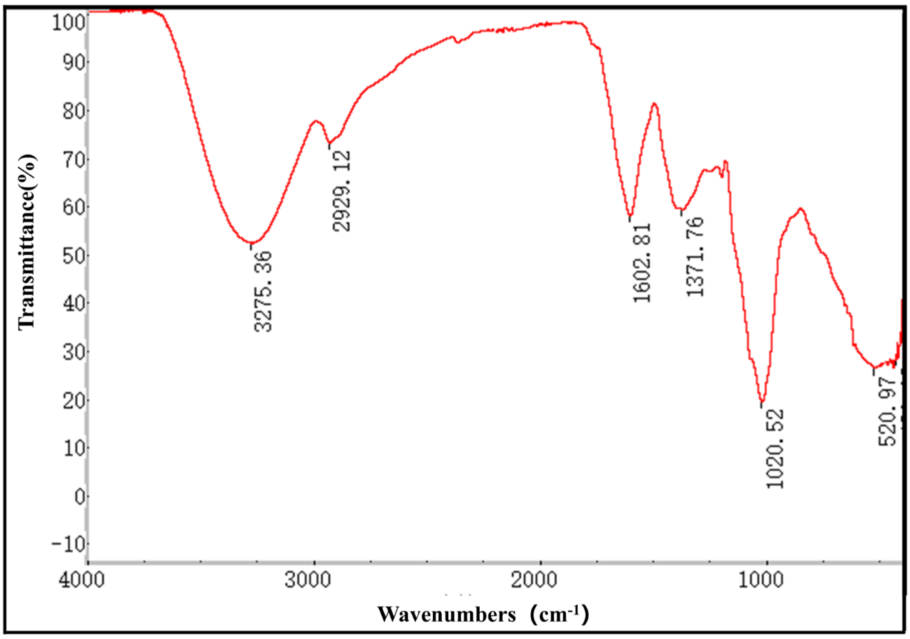

Figure S3. FTIR Spectrum of Poria cocos Polysaccharides (PCP). The FTIR spectrum of PCP shows characteristic absorption bands at 3275.36 cm^{-1} (O-H), 2929.12 cm^{-1} (C-H), 1602.81 cm^{-1}, 1371.76 cm^{-1}, 1020.52 cm^{-1} (C-O), and 520.97 cm^{-1}, confirming the polysaccharide nature of PCP. The absence of protein or nucleic acid bands in the 260-280 nm range supports the purity of the polysaccharide fraction.

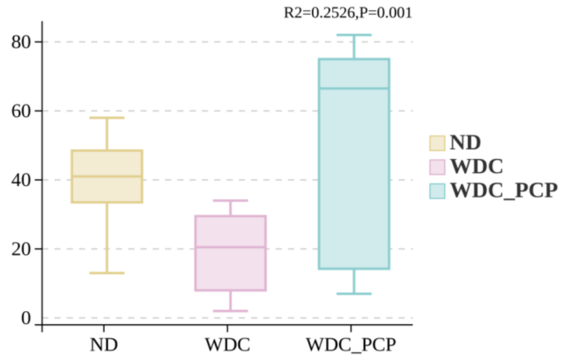

Figure S4. PERMANOVA Analysis of β-Diversity Using Unweighted_uniFrac Distances. A PERMANOVA test was applied to evaluate the differences in microbial community structure, yielding significant results with $R^2 = 0.2526$ and $p = 0.001$. The boxplots illustrate the median, interquartile range, and range (whiskers) of the unweighted UniFrac distances for each group, highlighting the distinct microbial structures associated with dietary and treatment conditions.

Table S1: Reagents used in the experiment

Reagent	Resource	Identifier
Carbon tetrachloride (CCl_4)	Sinopharm Chemical Reagent Co., Ltd., China	Cat#: 10006464
Corn oil	Sigma	Cat#: 8001-30-7
Ethyl Alcohol	FuYu Chemical, China	Cat#: 64-17-5
Dimethylbenzene	Guangzhou Chemical Reagent Factory, China	Cat#:1330-20-7
Haematoxylin staining solution	Biosharp, China	Cat#: BL702A
Eosin Y Solution	Solarbio, China	Cat#: G1100
Rhamsan gum	Sinopharm Chemical Reagent Co., Ltd., China	Cat#: 10004160
Glucose kit	Accu-Chek, Hong Kong	Cat#: L0140
Hepatic triglyceride (TG)	Nanjing Jiancheng Bioengineering Institute, China	Cat#: A110-1-1
Hepatic total cholesterol (TC)	Nanjing Jiancheng Bioengineering Institute, China	Cat#: A111-1-1
Serum alanine aminotransferase (ALT)	Nanjing Jiancheng Bioengineering Institute, China	Cat#: C009-2-1
Serum aspartate aminotransferase (AST)	Nanjing Jiancheng Bioengineering Institute, China	Cat#: C010-2-1
VeZol-Pure Total RNA Isolation Kit	Vazyme, America	Cat#: RC202-01
HiScript® II Q RT SuperMix for qPCR (+gDNA wiper)	Vazyme, America	Cat#: R223-01
Taq Pro Universal SYBR qPCR Master Mix（qPCR）	Vazyme, America	Cat#: Q712-02
Oil Red O Stain Kit	Nanjing Jiancheng Bioengineering Institute, China	Cat#: D027-1-1
Modified Sirius Red Stain Kit	Beijing Solarbio Technology CO., Ltd. China	Cat#G1472

Table S2: The primer sequence of the target gene used in the study

Gene name	Primer(forward)(5'-3')	Primer(Reverse)(5'-3')
GAPDH	TCAACAGCAACTCCCACTCTTCCA	TTGTCATTGAGAGCAATGCCAGCC
IL-1β	GAAATGCCACCTTTTGACAGTG	TGGATGCTCTCATCAGGACAG
IL-6	GTGACAACCACGGCCTTCCCTACT	GGTAGCTATGGTACTCCA
IL-18	ACAACTTTGGCCGACTTCAC	ATCAGTCTGGTCTGGGGTTC
TNF-α	GCGACGTGGAACTGGCAGAAG	GGTACAACCCATCGGCTGGCA
