# Supplementary figures and images for: Modulation of Gut Microbes and Hepatic Metabolites by PCP Ameliorates NASH and Fatigue-like Performance in Mice
Source: Nutrients. 2025 Dec 3;17(23):3797. doi: 10.3390/nu17233797 (PMC12693755; doi:10.3390/nu17233797)

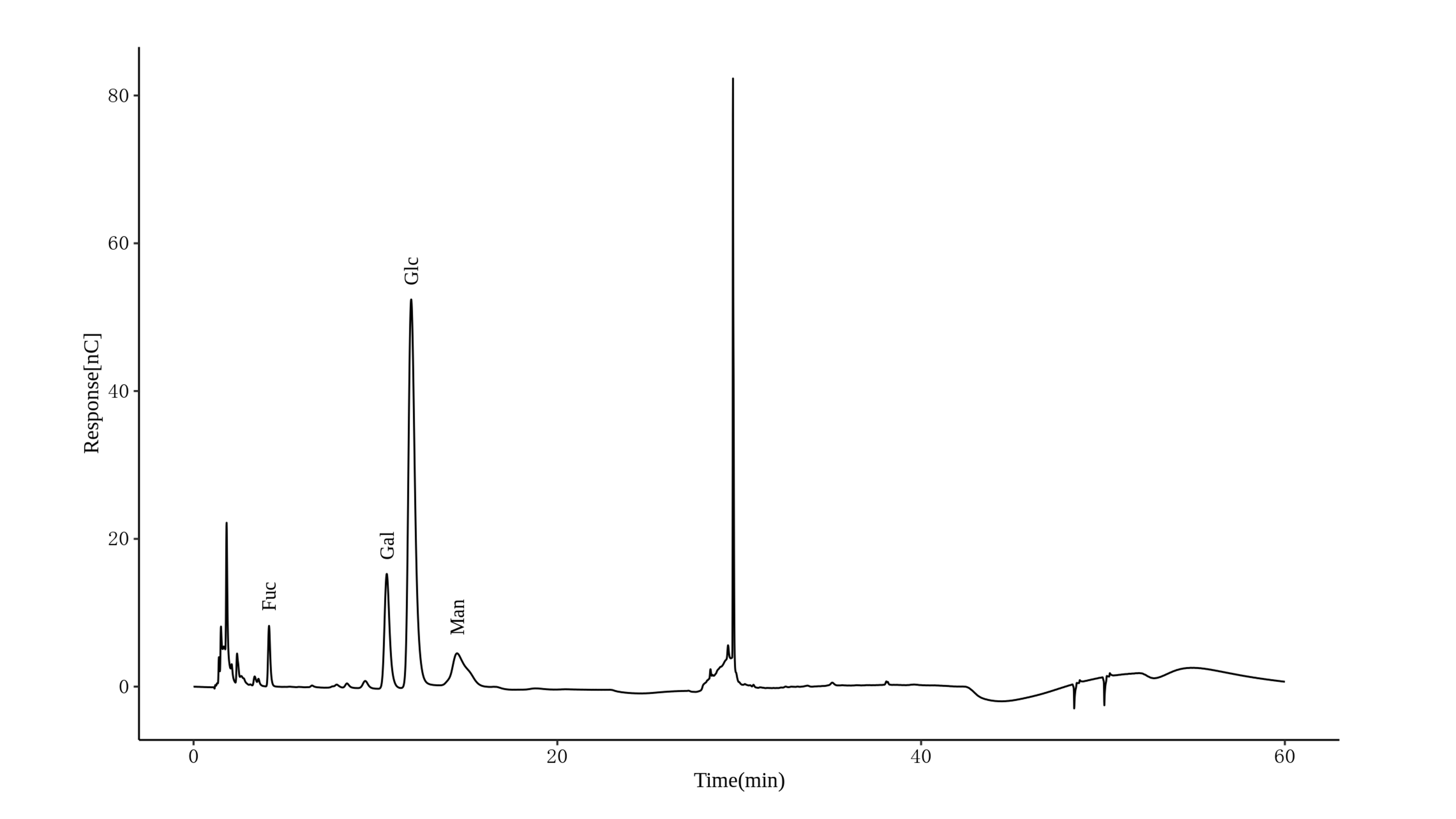

Supplement: Supplementary file 1 [file nutrients-17-03797-s001.zip › Supplementary material/Figure S1. Ion Chromatography Profile of Poria cocos Polysaccharides (PCP) Monosaccharides..TIF]

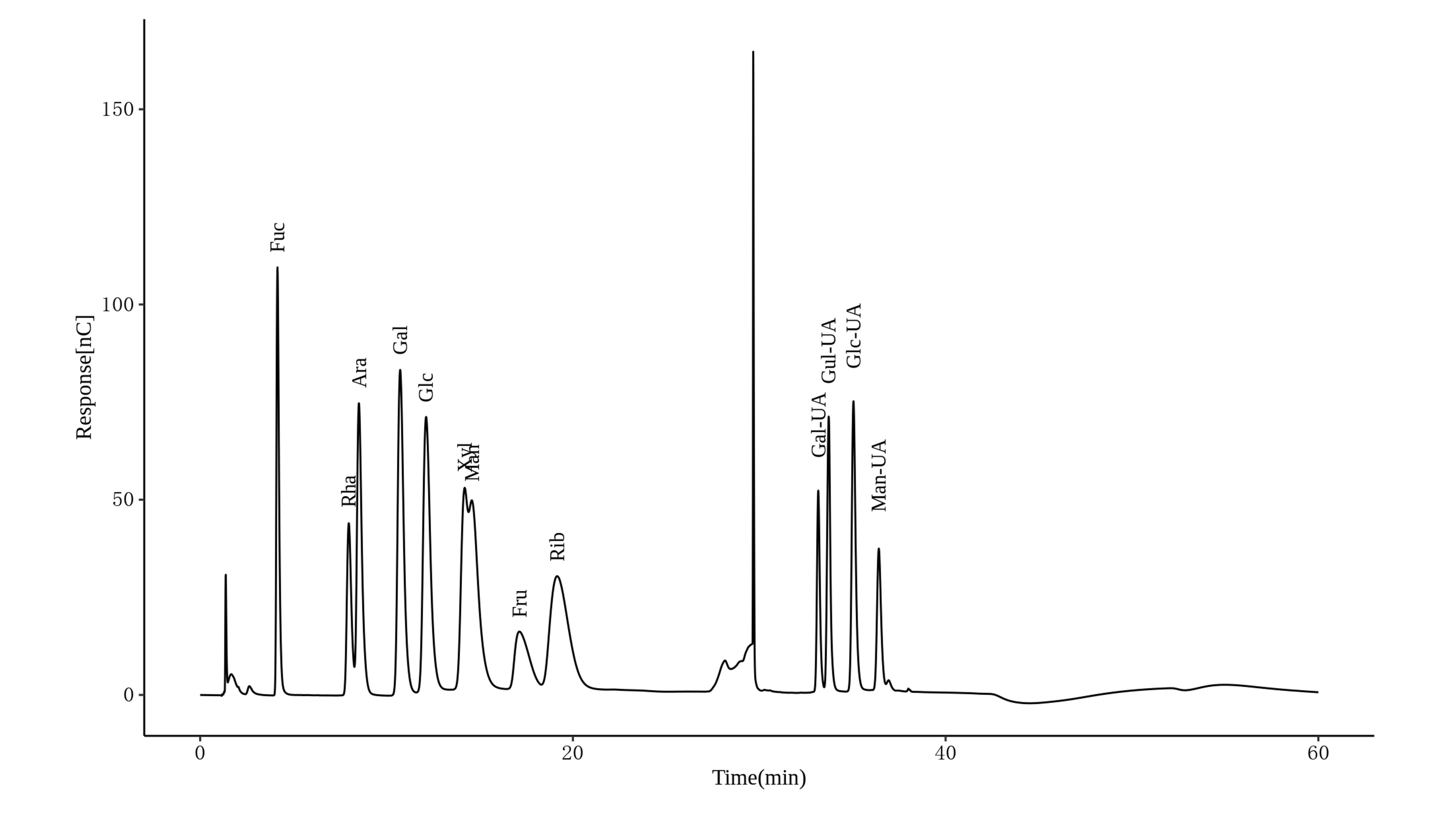

Supplement: Supplementary file 1 [file nutrients-17-03797-s001.zip › Supplementary material/Figure S2. Standard Monosaccharide Components Ion Chromatography..TIF]

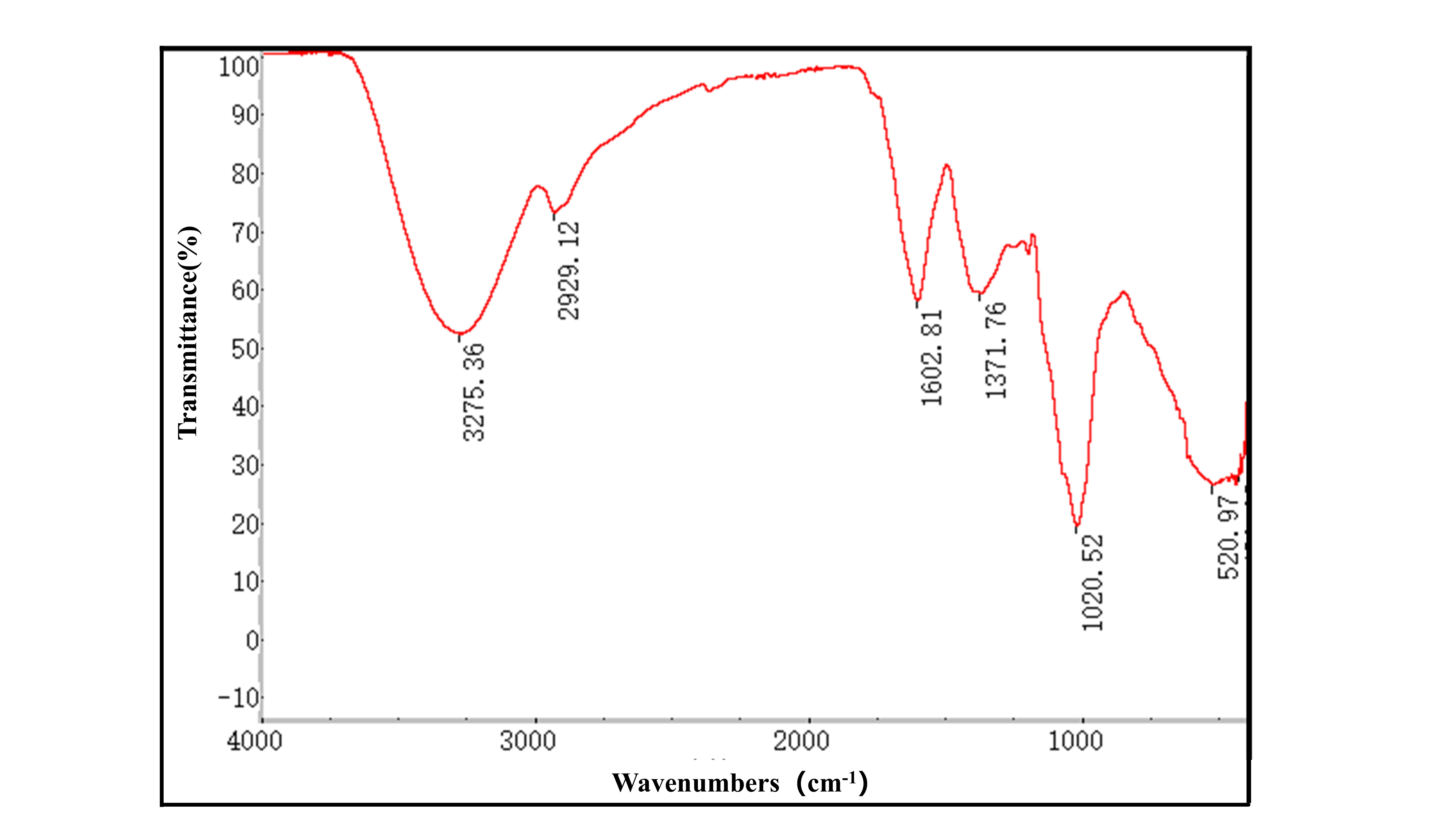

Supplement: Supplementary file 1 [file nutrients-17-03797-s001.zip › Supplementary material/Figure S3. FTIR Spectrum of Poria cocos Polysaccharides (PCP)..tif]

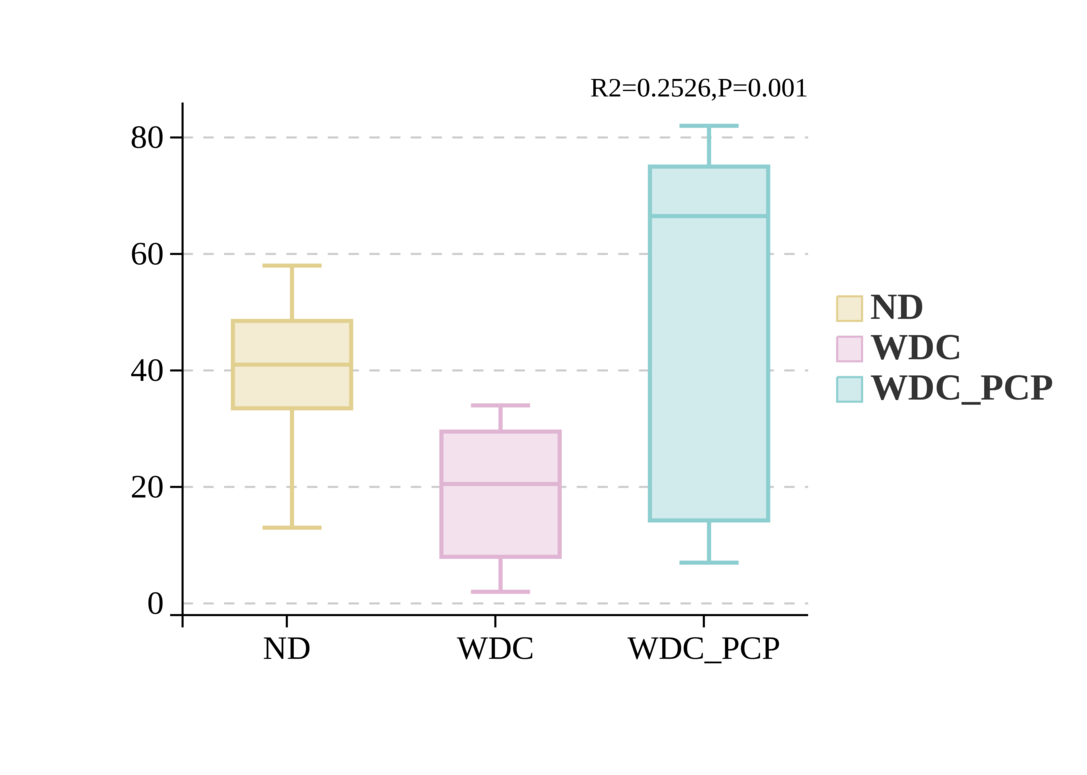

Supplement: Supplementary file 1 [file nutrients-17-03797-s001.zip › Supplementary material/Figure S4. PERMANOVA Analysis of a┬-Diversity Using Unweighted_uniFrac Distances..tiff]
